# Axl kinase drives immune checkpoint and chemokine signalling pathways in lung adenocarcinomas

**DOI:** 10.1186/s12943-019-0953-y

**Published:** 2019-02-11

**Authors:** Yoko Tsukita, Naoya Fujino, Eisaku Miyauchi, Ryoko Saito, Fumiyoshi Fujishima, Koji Itakura, Yorihiko Kyogoku, Koji Okutomo, Mitsuhiro Yamada, Tatsuma Okazaki, Hisatoshi Sugiura, Akira Inoue, Yoshinori Okada, Masakazu Ichinose

**Affiliations:** 1 Department of Respiratory Medicine, Tohoku University Graduate School of Medicine, Sendai, 980 8574 Japan; 2 Department of Anatomic Pathology, Tohoku University Graduate School of Medicine, Sendai, 980 8574 Japan; 3 Department of Palliative Medicine, Tohoku University School of Medicine, Sendai, 980 8574 Japan; 4 Department of Thoracic Surgery, Institute of Development, Aging and Cancer, Tohoku University, Sendai, 980 0872 Japan

**Keywords:** Non-small-cell lung cancer, Axl receptor tyrosine kinase, Immune checkpoint molecules, Chemokine signalling, Global gene expression array

## Abstract

Axl receptor tyrosine kinase is involved in the growth and metastasis and is an indicator of poor prognosis in several cancers including lung cancers. Although a mitogen-activated protein kinase (MAPK) pathway and an epithelial-to-mesenchymal transition (EMT) program are critical, molecular mechanisms underlying the Axl-driven cancer progression have not been fully elucidated. We aimed to identify molecules up-regulated by Axl kinase in lung adenocarcinomas. Through the global gene expression analysis and the functional annotation clustering, we found that *AXL* expression positively correlated with mRNA expressions of immune checkpoint molecules and chemokine receptors in non-small-cell lung cancers. Validation cohorts including our biobank confirmed that the *AXL* expression significantly correlated with expression of genes encoding programmed death-ligand1 (PD-L1) and CXC chemokine receptor 6 (CXCR6) in lung adenocarcinoma, especially in epidermal growth factor receptor (EGFR) mutation-positive adenocarcinoma. Pharmacological inhibition of Axl kinase activity decreased mRNA expressions of PD-L1 and CXCR6 in EGFR mutation-positive cell lines. Our data indicates the novel role of Axl kinase as a driver of immune checkpoint molecules and chemokine signalling pathways in the progression of lung adenocarcinomas. This study also highlights the necessity of clinical trials in order to test the efficacy of Axl kinase inhibition in the Axl-highly expressing subset of lung adenocarcinomas.

**Electronic supplementary material:**

The online version of this article (10.1186/s12943-019-0953-y) contains supplementary material, which is available to authorized users.

## Results and discussion

### Gene signatures of NSCLC with higher *AXL* mRNA expression

Activation of Axl receptor tyrosine kinase has a key role in the growth and metastasis of several cancers [1]. In lung adenocarcinomas, the protein expression of Axl and its ligand, growth arrest specific-6 (Gas6), is a critical indicator for the poor prognosis [2]. Moreover acquisition of Axl leads to resistance to epidermal growth factor receptor (EGFR)-targeted therapy for lung adenocarcinomas [3]. Based on these studies, the combination therapy of a selective Axl kinase inhibitor (BGB324) and an EGFR tyrosine kinase inhibitor (Erlotinib) for patients with Stage IIIB or IV non-small cell lung cancers (NSCLC) has currently been in a phase I/II clinical trial (NCT02424617).

Recent studies reported that intracellular kinases (e.g. mitogen-activated protein kinases, MAPKs) and epithelial-to-mesenchymal transition (EMT)-initiating transcription factors are involved in the Axl-driven survival and motility of cancers [1]. In addition a recent report indicates that Axl also up-regulates the expression of an immune checkpoint molecule, programmed death-ligand1 (PD-L1, or CD274) in head and neck cancers [4]. These studies suggest that the activation of Axl controls diverse molecular pathways contributing to a microenvironment beneficial to tumor progression. However the diverse array of molecules under Axl kinase has not been fully elucidated in lung cancer.

In order to characterise molecular phenotypes of NSCLC with higher *AXL* expression, we sought to identify genes whose expressions significantly correlated with *AXL* mRNA expression in a lung cancer tissue biobank (GSE accession number, GSE42127, *n* = 176 subjects, Additional file 1: Materials and Methods). In this discovery cohort, we found that 935 genes positively correlated with *AXL* expression (r_p_ > 0.4; Additional file 2: Table S1), whereas 137 genes were negatively correlated (r_p_ < − 0.4; Additional file 2: Table S2). A functional annotation clustering analysis revealed that gene ontology terms, “chemokine mediated signalling pathway” and “antigen processing and presentation”, were enriched in the 935 genes positively correlating with *AXL* mRNA expression (Additional file 2: Table S3). We failed to detect gene ontology terms in the 137 genes negatively correlating with *AXL* expression.

### Positive correlation of *AXL* expression with immune checkpoint molecules and chemokine receptors in lung adenocarcinomas

**Fig. 1:**
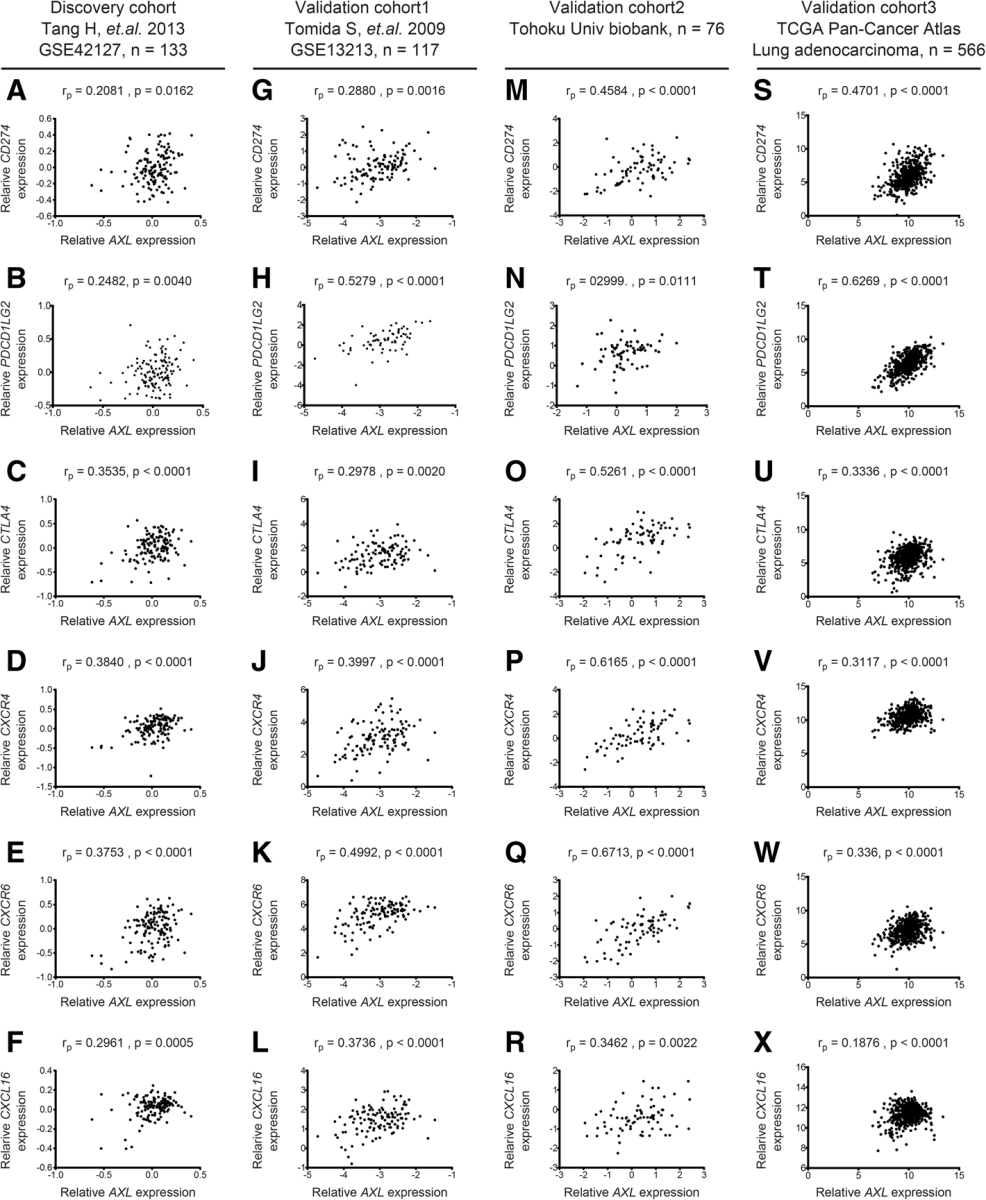
Genes encoding immune checkpoint molecules and chemokine/chemokine receptors were enriched in Axl-highly expressing NSCLC. Correlations of mRNA expressions between *AXL* and genes encoding PD-L1 (*CD274*; **a**, **g**, **m** and **s**), PD-L2 (*PDCD1LG2*; **b**, **h**, **n** and **t**), CTLA-4 (*CTLA4*; **c**, **i**, **o** and **u**), CXCR4 (*CXCR4*; **d**, **j**, **p** and **v**), CXCR6 (*CXCR6*; **e**, **k**, **q** and **w**) and CXCL16 (*CXCL16*; **f**, **l**, **r** and **x**). Microarray data from three cohorts (discovery cohort (**a**-**f**, GSE42127), validation cohort1 (**g**-**l**, GSE13213), validation cohort3 (**s**-**x**, TCGA Pan-Cancer Atlas) and qRT-PCR data from our cohort of Tohoku University Biobank (**m**-**r**) are shown. r_p_ is a Pearson correlation coefficient. The x-y axes indicate log2 transformed expression values of genes indicated

Our unbiased analysis of the discovery cohort microarray data suggests that chemokine signalling pathways and molecules associated with antigen processing and presentation (e.g., major histocompatibility complex (MHC) genes) are relevant to de novo *AXL*-highly expressing NSCLC. A recent report indicated that genes encoding MHC class I molecules positively correlated to PD-L1 mRNA expression in ovarian tumor cells [5]. Thus we sought to further validate the contribution of immune checkpoint molecules and chemokine signalling pathways to Axl-expressing adenocarcinomas. We have established Tohoku University Biobank (Additional file 2: Table S4). Using three independent validation cohorts of lung adenocarcinomas (Validation cohort1: GSE13213 in the Gene Expression Omnibus (GEO) of the National Center for Biotechnology Information (NCBI), *n* = 117; Validation cohort2: Our cohort of Tohoku University Biobank, *n* = 76; Validation cohort3: Lung Adenocarcinoma in The Cancer Genome Atlas (TCGA), Pan-Cancer Atlas, *n* = 566), we found that *AXL* expression significantly correlated with the expressions of genes encoding immune checkpoint molecules (*CD274*, *PDCD1LG2* and *CTLA4*), chemokine receptors (*CXCR4* and *CXCR6*) or a chemokine (*CXCL16*) in the discovery cohort as well as the three validation cohorts (Fig. 1). These chemokine receptors have been attributed to the invasion and metastases of cancers [6].

Higher expression of genes encoding PD-L1, CXCR6 and CXCL16 in Axl-expressing adenocarcinomas with mutated EGFR.

**Fig. 2:**
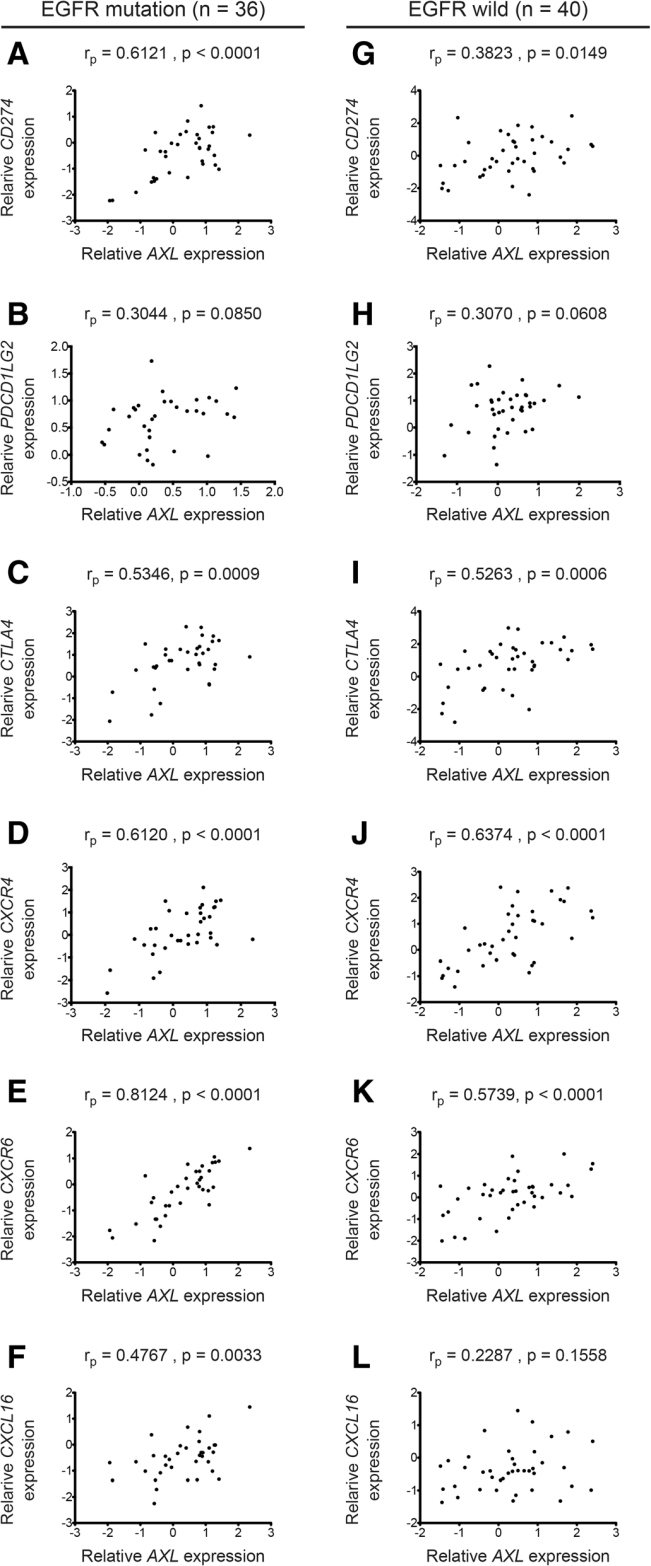
Genes encoding PD-L1 and CXCR6 correlated with *AXL* mRNA in EGFR-mutated lung adenocarcinomas. Correlations of mRNA expressions between *AXL* and genes encoding PD-L1 (*CD274*; **a** and **g**), PD-L2 (*PDCD1LG2*; **b** and **h**), CTLA-4 (*CTLA4*; **c** and **i**), CXCR4 (*CXCR4*; **d** and **j**), CXCR6 (*CXCR6*; **e** and **k**) and CXCL16 (*CXCL16*; **f** and **l**) in EGFR mutation-positive (**a**-**f**) and EGFR wild-type (**l**-**l**) lung adenocarcinomas from Tohoku University Biobank. r_p_ is a Pearson correlation coefficient. The x-y axes indicate log2 transformed expression values of genes indicated

To determine whether the EGFR mutation status is influential in the correlation of *AXL* expression with the immune checkpoint molecules and the chemokine/chemokine receptors, we evaluated Pearson correlation coefficients between EGFR mutation-positive and wild type lung adenocarcinomas in our cohort of Tohoku University Biobank. We found higher correlations of gene expressions between *AXL* and three genes (*CD274*, *CXCR6* and *CXCL16*) in EGFR-mutation positive adenocarcinomas than in wild type (Fig. 2).

### Pharmacological inhibition of Axl kinase activity decreases the mRNA expressions of *CD274, PDCD1LG2* and *CXCR6* in EGFR mutation-positive lung adenocarcinoma cell lines

**Fig. 3:**
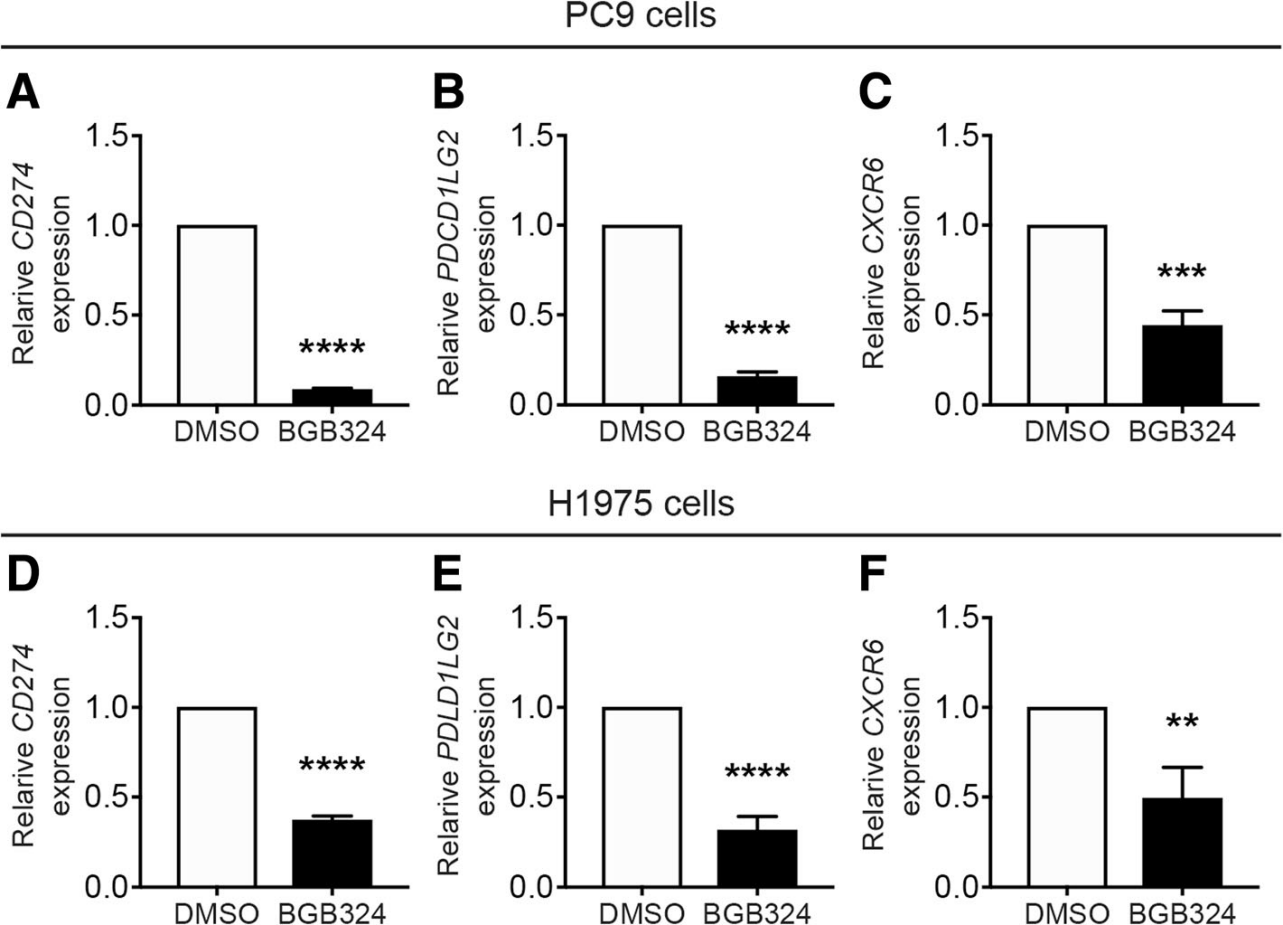
A selective inhibitor of Axl kinase decreased PD-L1, PD-L2 and CXCR6 mRNA expression in vitro. Relative expression values of *CD274* (PD-L1; **a**, **d**), *PDCD1LG2* (PD-L2; **b**, **e**) and *CXCR6* (**c**, **f**) in PC9 cells (**a**-**c**) and H1975 cells (**d**-**f**) six hours after treatment with 0.1% DMSO or Axl inhibitor BGB324 (10 μM). Each value represents the fold-increase in gene expression of BGB324-treated cells compared to that of DMSO-treated cells in each experiment. Data are expressed as the mean ± SD of three independent experiments. ****p < 0.0001, **p < 0.01, *p < 0.05, vs. DMSO; two-tailed paired t-test. NS indicates not significant

To determine whether and how Axl regulates the mRNA expressions of PD-L1, PD-L2 and CXCR6 in EGFR-mutated lung adenocarcinomas, we treated two EGFR mutation-positive lung adenocarcinoma cell lines (PC9 cells and H1975 cells) with a selective Axl kinase inhibitor BGB324 or with siRNA targeting Axl. We confirmed mRNA and protein expressions of Axl in both PC9 cells and H1975 cells (data not shown) and verified that the Axl inhibitor BGB324 decreased phosphorylation of Axl in these cell lines in a dose-dependent manner (Additional file 3: Figure S1). We found that BGB324 significantly decreased the expressions of *CD274* and *PDCD1LG2* and *CXCR6* in both cell lines (Fig. 3). This was confirmed by siRNA-mediated Axl silencing (Additional file 3: Figure S2). We further confirmed that BGB324 reduced phosphorylation of extracellular signal-regulated kinase (ERK) and AKT in PC9 cells and H1975 cells (Additional file 3: Figure S3). A selective MEK inhibitor (U1206) or an allosteric AKT inhibitor (MK-2206) decreased mRNA expression of PD-L1 (Additional file 3: Figure S4), suggesting that ERK and AKT were at least partly involved in downstream signalling pathways of Axl [7].

### Positive correlation of *AXL* expression with co-inhibitory and co-stimulatory molecules across multiple cancers

So far, through the unbiased transcriptome analyses and the subsequent in vitro cell culture assays, we identified a new role of Axl kinase in up-regulating PD-L1 in lung adenocarcinoma. We then sought to extend this finding to several cancers including EGFR-related cancers (glioblastoma and colon cancer) and other solid tumors (melanoma, renal cell carcinoma, gastric cancer and head and neck cancer), most of which Nivolumab have been approved for the treatment. In addition we wished to clarify a broader expression pattern of co-inhibitory and co-stimulatory molecules whose expressions correlated with Axl kinase. Using TCGA datasets, we found that the EGFR-related cancers exhibited more significant correlation of PD-L1 mRNA expression with *AXL* expression (Additional file 2: Table S6). Moreover co-inhibitory receptors (*PDCD1* encoding PD-1 and *HAVCR2* encoding Tim3) and co-stimulatory receptors (*CD28* and *CD137*) showed the significant correlation with *AXL* across a variety of cancers (Additional file 2: Table S6).

In summary, our findings suggest that Axl kinase up-regulates several pathways such as immune checkpoint molecules/chemokine signalling involved in regulation of the immune microenvironment and tumor proliferation (Additional file 3: Figure S5). Considering the diverse non-genomic actions driven by Axl, monotherapies targeting immune checkpoint molecules such as PD-L1 or PD-L2/PD-1 or chemokines might not yield sufficient treatment efficacy in patients with Axl-positive NSCLC. This hypothesis is supported by a recent report demonstrating that Axl was up-regulated in melanomas with innate resistance to an anti-PD-1 therapy [8]. Given our results, it might be possible that Axl kinase inhibition could increase the sensitivity to a PD-1 targeted therapy. In addition NSCLC harbouring EGFR mutations are associated with poor efficacy of PD-1/PD-L1 inhibitors [9]. We therefore speculate that Axl kinase drives multiple molecular pathways promoting tumor progression especially in EGFR-mutated lung adenocarcinoma.

There are some limitations in the present study. First we did not observe correlation between Axl kinase and these immune-related molecules at a protein level. Second we have not tested if Axl kinase promotes immune checkpoint molecules and chemokines that function in in vivo settings. Third we cannot exclude a possibility that PD-L1-bearing cell-types other than cancer cells were involved in this bioinformatic analysis as we analyzed transcriptome data from whole tissue lysates but not at a single cell level. Further validation studies using immunohistochemistry, animal models and a single cell-RNA sequence are required.

In conclusion, Axl-highly expressing lung adenocarcinomas exhibit higher expressions of multiple genes encoding immune checkpoint molecules and chemokines/chemokine receptors. Our data therefore provide a rationale for further mechanistic studies to validate the role of Axl kinase as a driver in diversifying downstream molecules in NSCLC and, ultimately, to perform trials testing the efficacy of Axl inhibition in the Axl-highly expressing subset of NSCLC.

## Additional files

Additional file 1: Materials and Methods. (DOCX 37 kb)

Additional file 2: **Table S1.** 935 genes positively correlating *AXL* mRNA expression in a NSCLC biobank (GSE accession number, GSE42127). **Table S2.** 137 genes negatively correlating *AXL* mRNA expression in a NSCLC biobank (GSE accession number, GSE42127). **Table S3.** Ontology terms enriched in the 935 genes that positively correlated *AXL* mRNA expression in a lung cancer biobank (GSE accession number, GSE42127). **Table S4.** Characteristics of patients (*n* = 76) with lung adenocarcinomas of our Tohoku University Biobank for the qPCR validation study. **Table S5.** Primer list. **Table S6.** Correlation of mRNA expression between *AXL* and genes encoding co-stimulatory/inhibitory molecules as shown by Pearson’s r. (XLSX 68 kb)

Additional file3: **Figure S1.** A dose-dependent decrease in phosphorylation of Axl in PC9 cells by the Axl kinase inhibitor. **Figure S2.** Knockdown of Axl decreases mRNA expression of PD-L1, PD-L2 and CXCR6 in vitro. **Figure S3.** Immunoblots indicating decreases in phosphorylation of ERK1/2 and AKT by the Axl kinase inhibitor. **Figure S4.** A selective MEK1/2 inhibitor or an AKT inhibitor reduces PD-L1 mRNA expression in vitro. **Figure S5**. Diverse downstream pathways driven by Axl receptor tyrosine kinase in non-small-cell lung cancer. (DOCX 2506 kb)

## Abbreviations

CXCL: CXC chemokine ligand
CXCR: CXC chemokine receptor
EGFR: Epidermal growth factor receptor
EMT: Epithelial-to-mesenchymal transition
ERK: Extracellular signal-regulated kinase
Gas6: Growth arrest specific-6
GEO: The Gene Expression Omnibus
MAPKs: Mitogen-activated protein kinases
MHC: Major histocompatibility complex
NCBI: The National Center for Biotechnology Information
NSCLC: Non-small-cell lung cancer
PD-L1: Programmed death-ligand1
PI3K: Phosphoinositide 3-kinase
TCGA: The Cancer Genome Atlas
