# Supplementary material for: Axl kinase drives immune checkpoint and chemokine signalling pathways in lung adenocarcinomas
Source: Mol Cancer. 2019 Feb 11;18:24. doi: 10.1186/s12943-019-0953-y (PMC6369543; doi:10.1186/s12943-019-0953-y)
Supplement: Supplementary file 1 — Materials and Methods. (DOCX 37 kb) [file 12943_2019_953_MOESM1_ESM.docx]

**Materials and Methods**

**Microarray analysis**

Global gene expression array data of human lung cancers were downloaded from the NCBI Gene Expression Omnibus (http://www.ncbi.nlm.nih.gov/geo; GEO Series accession numbers, GSE42127 [1] and GSE13213 [2]) or the cBioPortal for Gancer Genomics [3,4]. Statistics for gene expression were computed using Subio Platform (Subio Inc., Amami, Japan) or on the cBioPortal. Gene ontology term enrichment was analysed by the Functional Annotation Clustering Tool in the Database for Annotation, Visualization and Integrated Discovery (DAVID V.6.8, http://david.abcc.ncifcrf.gov) [5]. The major gene ontology terms associated with each group were manually summarised based on gene-term enrichment buttons provided for each functional group [6].

**Patients and preparation of human lung cancer tissues**

We have established Tohoku University Biobank (Additional file 2: Table S4). The lung tissues were provided by patients who underwent lung resection at the Department of Thoracic Surgery at Tohoku University Hospital between August 2014 and November 2016. Those with radiation therapy were excluded from the study. This study was approved by the Ethnic Committee at Tohoku University School of Medicine. Written informed consents were received from all patients before the surgery. Lung specimens were harvested immediately after surgical resection and submerged in RNA*later* Stabilization Solution (Thermo Fishier Scientific, Waltham, MA, USA). Specimens were stored with RNA*later* at −80 °C. Thorough pathological examination of resected lung tissue was performed and findings recorded in accordance with the seventh edition of the Tumour Node Metastasis (TNM) Lung Cancer Staging classification.

**Cell culture**

EGFR mutant human lung adenocarcinoma cell lines PC9 (exon 19 deletion) and H1975 (L858R and T790M) were purchased from Immuno-Biological Laboratories Co. (Takasaki, Gunma, Japan). Cell lines were cultured in growth medium consisting of RPMI (Sigma, St Louis, MO, USA) supplemented with 10% foetal calf serum (FCS, Nichirei, Tokyo, Japan), 100 unit/mL penicillin and 100 μg/mL streptomycin (Thermo Fishier Scientific) in a humidified 5% CO_2_ incubator at 37 °C.

PC9 cells or H1975 cells were plated at 2.5×10^5^ cells/well on 6-well plates. Two days after the plating the growth medium was replaced with RPMI + 10 % FCS + penicillin/streptomycin containing either 0.1% dimethyl sulfoxide (DMSO, Sigma), a selective small molecule inhibitor for Axl kinase BGB324 (10 μM, Selleckchem, Houston, TX, USA), a MEK1/2 inhibitor U0126 (10 μM, Calbiochem, La Jolla CA, USA) or an allosteric AKT inhibitor MK-2206 (10 μM, Cayman Chemical, Ann Arbor, Michigan, USA). After six hours, cells were harvested for an RNA extraction procedure or a western blot.

**siRNA-mediated knockdown**

Knockdown of Axl was conducted by specific single siRNA using Silencer Select siRNA for Axl (Thermo Fisher Scientific #s1845). Silencer Select Negative control #1 siRNA (Thermo Fisher Scientific) was used as non-targeted siRNA. Lipofectamine RNAiMAX (Thermo Fisher Scientific) was used to introduce siRNAs to cell lines used. Transient transfection was performed according to the manufacturer’s instruction. For the evaluation of knockdown efficacy, PC-9 cells or H1975 cells were seeded at a density of 40 000 cells per well on a 12-well format plate in RPMI + 10% FCS. After 24 h, cells were transfected with siRNA-lipofectamine complexes and harvested three days after the transfection.

**RNA extraction and quantitative real-time PCR**

Total RNA was extracted from cultured cells or surgical specimens using RNeasy Mini Kit (Qiagen, Valencia, CA, USA) or TRIzol Reagent (Thermo Fisher Scientific), respectively as per the manufacturer’s instructions. Total RNA of surgical specimens was further purified using RNeasy Mini Kit (Qiagen). cDNA was synthesised using High Capacity RNA-to-cDNA Kit (Thermo Fisher Scientific).　Quantitative real-time PCR was performed using SYBR Green PCR Master Mix (Thermo Fisher Scientific) and detection was executed with StepOne Plus Real-Time PCR System (Thermo Fisher Scientific) in duplicate. The relative expression levels of the specific mRNA were calculated using the 2^−ΔΔCt^ method. The primers for *AXL*, *B2M* and *RPLP0* were purchased from Takara Bio Inc. (Shiga, Japan). The other primers (*CD274*, *PDCD1LG2*, *CTLA4*, *CXCR4*, *CXCR6* and *CXCL16*) synthesised as per PrimerBank (https://pga.mgh.harvard.edu/primerbank/) shown in Additional file 2, Table S5. *B2M* and *RPLP0* were used as internal controls.

**Enzyme-Linked Immunosorbent Assay (ELISA) for total Axl (t-Axl) and phosphorylated Axl (p-Axl)**

An Axl kinase inhibitor, BGB324, was serially diluted with RPMI + 10% FCS + penicillin/streptomycin at concentrations indicated. PC9 cells were plated at 2.5×10^5^ cells/well on six-well plates. Two days after the plating, the growth medium was replaced with RPMI + 10% FCS + penicillin/streptomycin containing either 0.1% DMSO or a BGB324 (0.01-10 μM). After three hours, we collected cell lysates and then measured the amounts of t-Axl and p-Axl by ELISA as previously described [7].

**Western Blot**

To evaluate the phosphorylation of ERK1/2 and AKT, cells were incubated with either 0.1% DMSO, BGB324 (10 μM), U1206 (10 μM) or MK-2206 (10 μM) for 30 minutes. Western blot was performed as previously described with some modification [8]. Briefly, a membrane was blocked for two hours with 5% non-fat dry milk in Tris-buffered saline with 0.05% Tween-20 (TBST) except for an antibody against phosphorylated Axl or with *Can Get Signal*^®^ Immunoreaction Enhancer Solution (TOYOBO CO., LTD, Osaka, Japan) for detecting phosphorylated Axl, and then incubated with the following primary antibodies overnight at 4 °C: rabbit anti-human p-ERK1/2 antibody (Cell Signaling Technology, Danvers, MA, USA, #9101, 1:1000); rabbit anti-human t-ERK1/2 (Cell Signaling Technology, #4695, 1:5000); rabbit anti-human p-AKT antibody (Cell Signaling Technology, #4060, 1:2000); rabbit anti-human t-AKT antibody (Cell Signaling Technology, #4691, 1:1000); rabbit anti-human p-Axl (R&D systems, Minneapolis, MN, USA, #AF2228, 1:200); rabbit anti-human t-Axl antibody (Cell Signaling Technology, #8661, 1:1000) anti β-actin antibody (Sigma, #A5316, 1:5000). The membrane was washed three times and incubated with horseradish peroxidase (HRP)-conjugated secondary antibodies for one hour at room temperature.

**Statistical analysis**

All statistical analyses were performed using GraphPad Prism V.6 (GraphPad Software, Inc., San Diego, California, USA). To test correlations between two datasets, a Pearson correlation coefficient (r_p_) and the p-value were calculated. The data are expressed as the means ± standard deviation (SD). Two-tailed paired student’s test was used to compare the statistical difference between two groups. Probability values < 0.05 were considered significant.

**References**

1. Tang H, Xiao G, Behrens C, Schiller J, Allen J, Chow C-W, et al. A 12-gene set predicts survival benefits from adjuvant chemotherapy in non-small cell lung cancer patients. Clin. Cancer Res. 2013;19:1577–86.

2. Tomida S, Takeuchi T, Shimada Y, Arima C, Matsuo K, Mitsudomi T, et al. Relapse-related molecular signature in lung adenocarcinomas identifies patients with dismal prognosis. J. Clin. Oncol. 2009;27:2793–9.

3. Cerami E, Gao J, Dogrusoz U, Gross BE, Sumer SO, Aksoy BA, et al. The cBio cancer genomics portal: an open platform for exploring multidimensional cancer genomics data. Cancer Discov. American Association for Cancer Research; 2012;2:401–4.

4. Gao J, Aksoy BA, Dogrusoz U, Dresdner G, Gross B, Sumer SO, et al. Integrative analysis of complex cancer genomics and clinical profiles using the cBioPortal. Sci Signal. 2013;6:pl1–pl1.

5. Huang DW, Sherman BT, Lempicki RA. Systematic and integrative analysis of large gene lists using DAVID bioinformatics resources. Nat Protoc. 2009;4:44–57.

6. Huang DW, Sherman BT, Lempicki RA. Bioinformatics enrichment tools: paths toward the comprehensive functional analysis of large gene lists. Nucl. Acids Res. 2009;37:1–13.

7. Fujino N, Kubo H, Maciewicz RA. Phenotypic screening identifies Axl kinase as a negative regulator of an alveolar epithelial cell phenotype. Lab. Invest. 2017;97:1047–62.

8. Numakura T, Sugiura H, Akaike T, Ida T, Fujii S, Koarai A, et al. Production of reactive persulfide species in chronic obstructive pulmonary disease. Thorax. 2017;72:1074–83.
