# Supplementary material for: Axl kinase drives immune checkpoint and chemokine signalling pathways in lung adenocarcinomas
Source: Mol Cancer. 2019 Feb 11;18:24. doi: 10.1186/s12943-019-0953-y (PMC6369543; doi:10.1186/s12943-019-0953-y)
Supplement: Supplementary file 3 — Figure S1. A dose-dependent decrease in phosphorylation of Axl in PC9 cells by the Axl kinase inhibitor. Figure S2. Knockdown of Axl decreases mRNA expression of PD-L1, PD-L2 and CXCR6 in vitro. Figure S3. Immunoblots indicating decreases in phosphorylation of ERK1/2 and AKT by the Axl kinase inhibitor. Figure S4. A selective MEK1/2 inhibitor or an AKT inhibitor reduces PD-L1 mRNA expression in vitro. Figure S5. Diverse downstream pathways driven by Axl receptor tyrosine kinase in non-small-cell lung cancer. (DOCX 2506 kb) [file 12943_2019_953_MOESM3_ESM.docx]

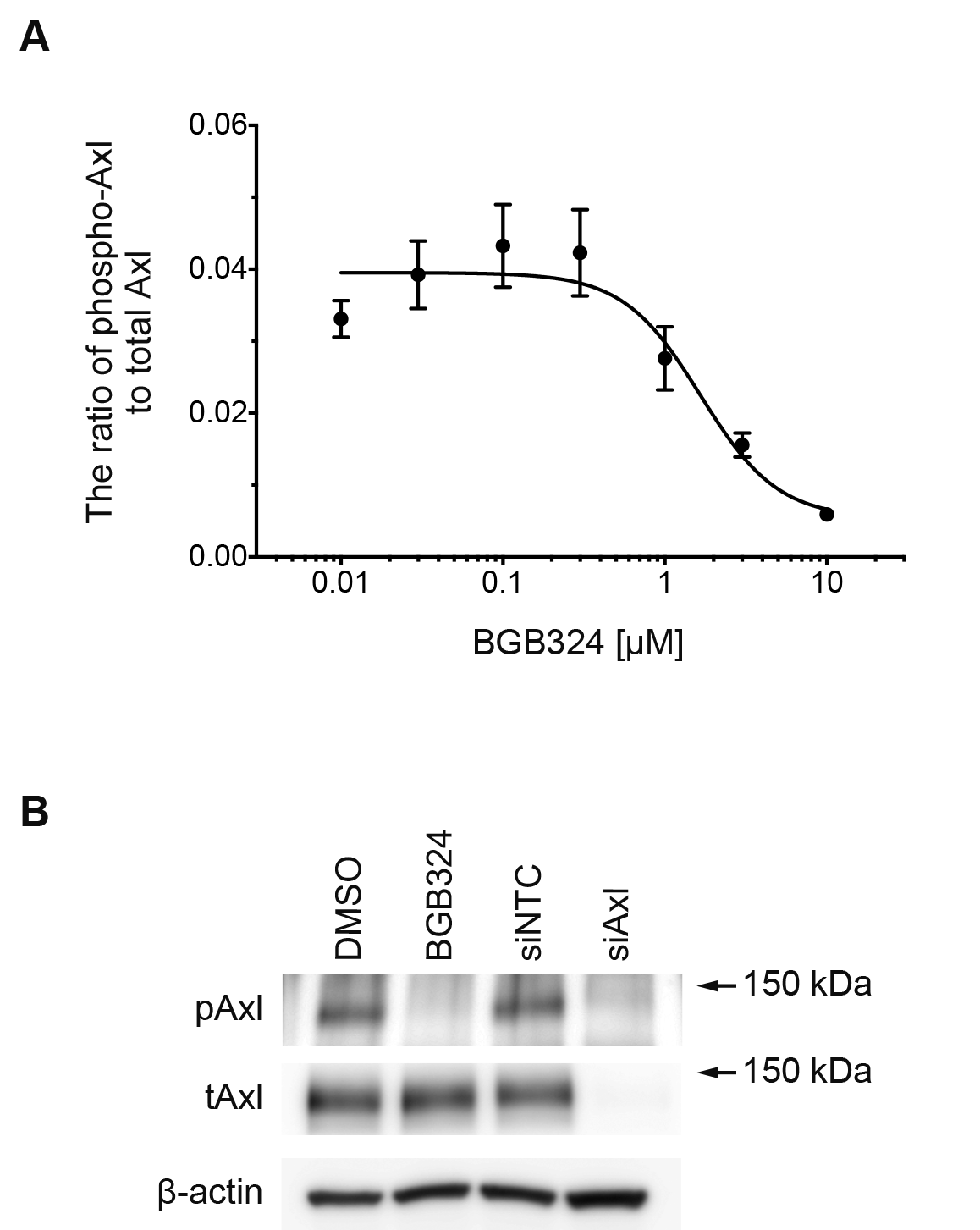
**Figure S1:** (A) A dose-dependent decrease in the ratio of phospho-Axl to total Axl in PC9 cells treated with the Axl kinase inhibitor BGB324. Data are mean ± SD of three independent experiments. The x axis indicates concentration of BGB324 in a log scale. (B) Western blots for phosphorylated Axl (pAxl) and total Axl (tAxl) in PC9 cells treated with medium containing 0.1% DMSO, BGB324 at 10μM, non-targeted siRNA (siNTC) and siRNA for Axl (siAxl). β-actin was used as an endogenous control.

**Figure S2: Knockdown of Axl decreases mRNA expression of PD-L1, PD-L2 and CXCR6 *in vitro*.** Relative expression values of *CD274* (PD-L1; A, D), *PDCD1LG2* (PD-L2; B, E) and *CXCR6* (C, F) in PC9 cells (A-C) and H1975 cells (D-F) three days after transfection of non-targeted siRNA (siNTC) or siRNA targeting Axl (siAxl). Data are expressed as the mean ± SD of triplicate cultures. **p < 0.01, *p < 0.05, vs. siNTC; two-tailed paired t-test. NS indicates not significant.

**
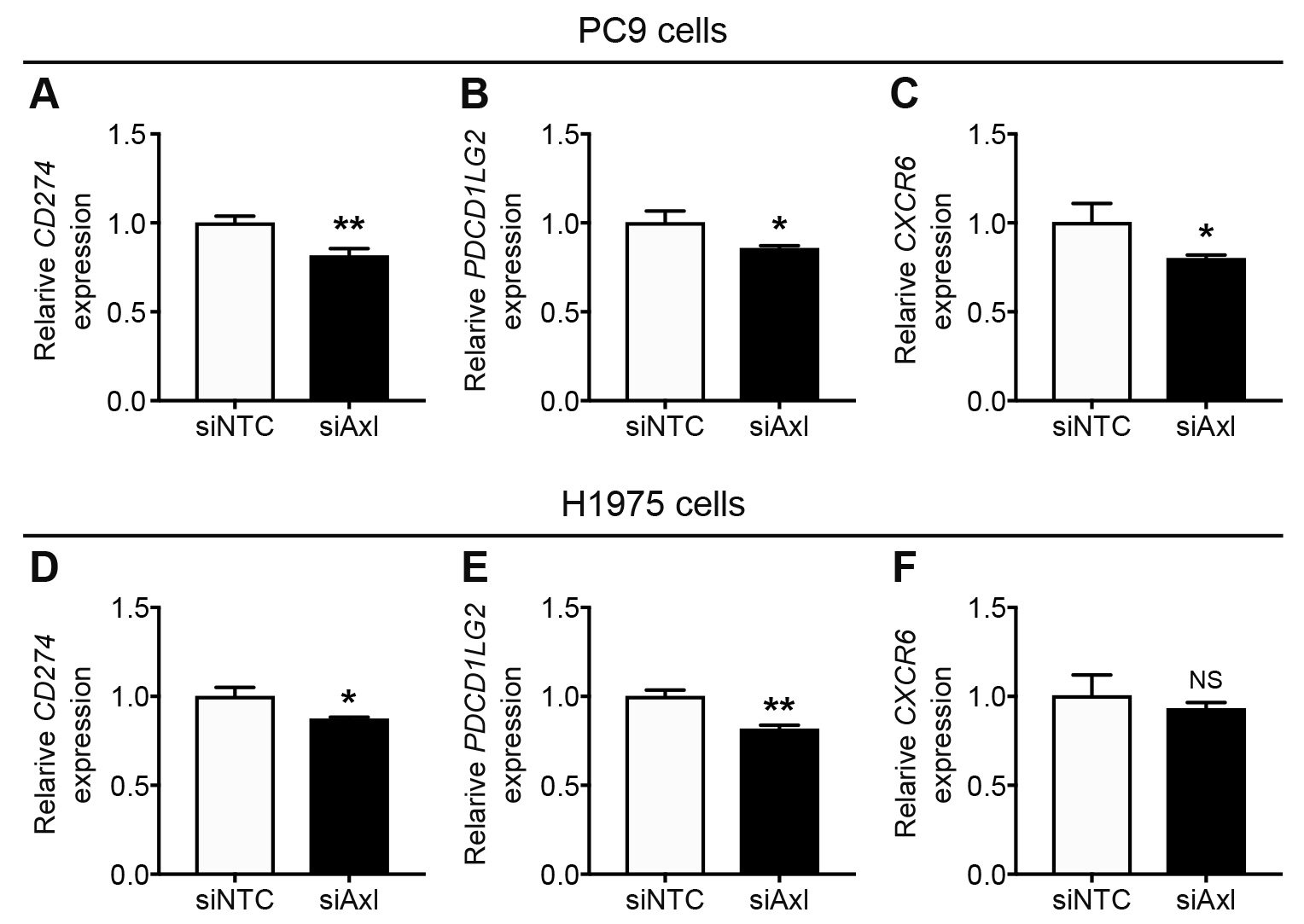
**

**
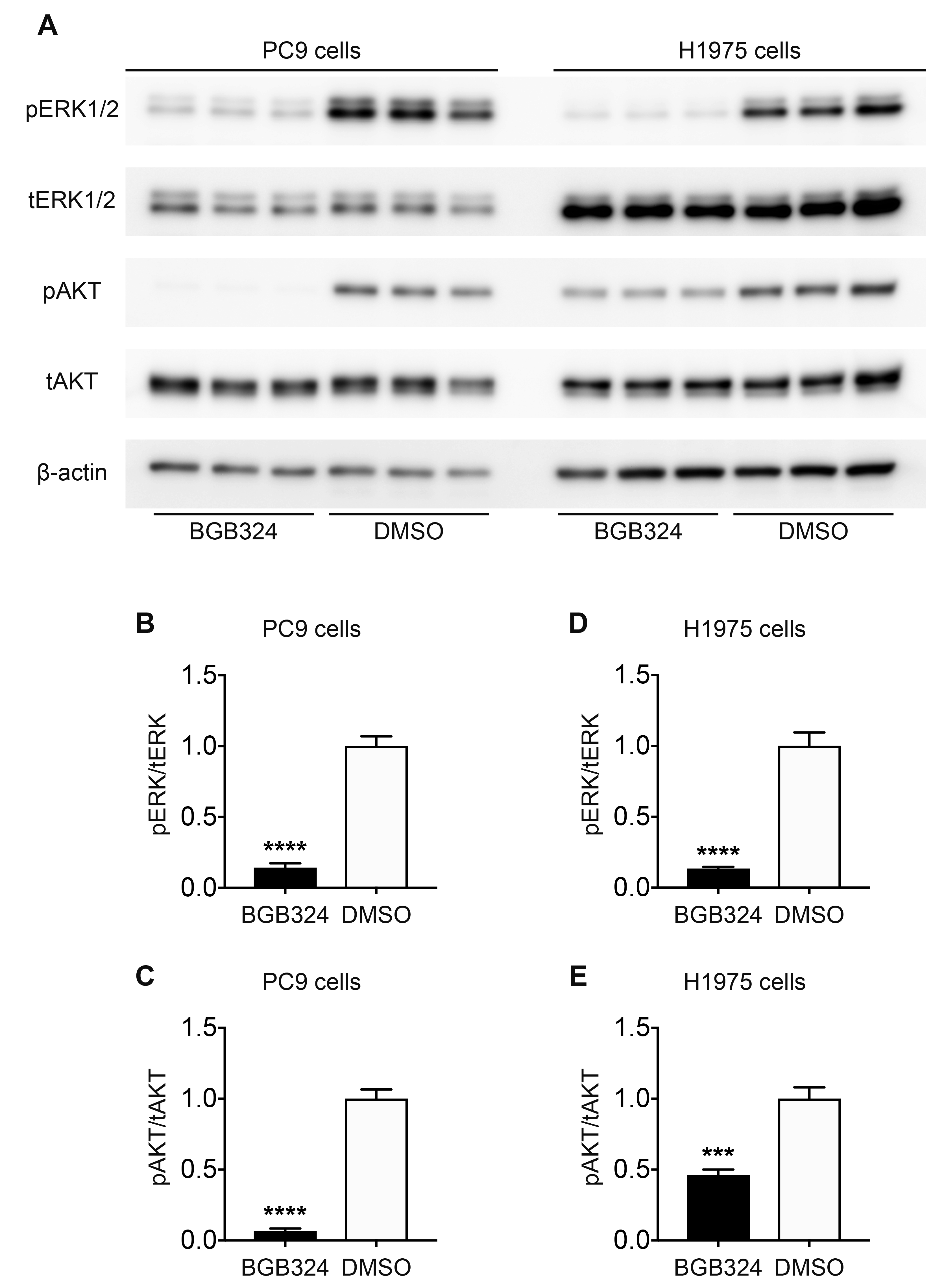
Figure S3: Decreases in phosphorylation of ERK1/2 and AKT by the Axl kinase inhibitor BGB324.** (A) Immunoblots for phosphorylated ERK1/2 (pERK1/2), total ERK1/2 (tERK1/2), phosphorylated AKT (pAKT) and total AKT (tAKT) with BGB324 (10 μM) and 0.1%DMSO. β-actin served as an internal control. (B-D) Quantification of the decrease in the phosphorylation of ERK1/2 or AKT in PC9 cells or H1975 cells. Data are mean ± SD of triplicate cultures. ****p < 0.0001, ***p < 0.001, vs. DMSO; two-tailed paired t-test.

**Figure S4: A selective MEK1/2 inhibitor (U1206) or an AKT inhibitor (MK-2206) reduces PD-L1 mRNA expression *in vitro*.** (A-D) Immunoblots for phosphorylated ERK1/2 (pERK1/2), total ERK1/2 (tERK1/2), phosphorylated AKT (pAKT) and total AKT (tAKT) in PC9 cells (A, B) and H1975 cells (C, D) treated with a selective MEK1/2 inhibitor (U1206, 10 μM; A, C) or an allosteric AKT inhibitor (MK-2206, 10 μM; B, D). β-actin serves as an internal control. (E-H) Relative mRNA expression of *CD274* encoding PD-L1 in PC9 cells (E, F) and H1975 cells (G, H) treated with U1206 (E, G) or MK-2206 (F, H). Data are mean ± SD of triplicate cultures. ****p < 0.0001, **p < 0.01, vs. DMSO; two-tailed paired t-test.

**
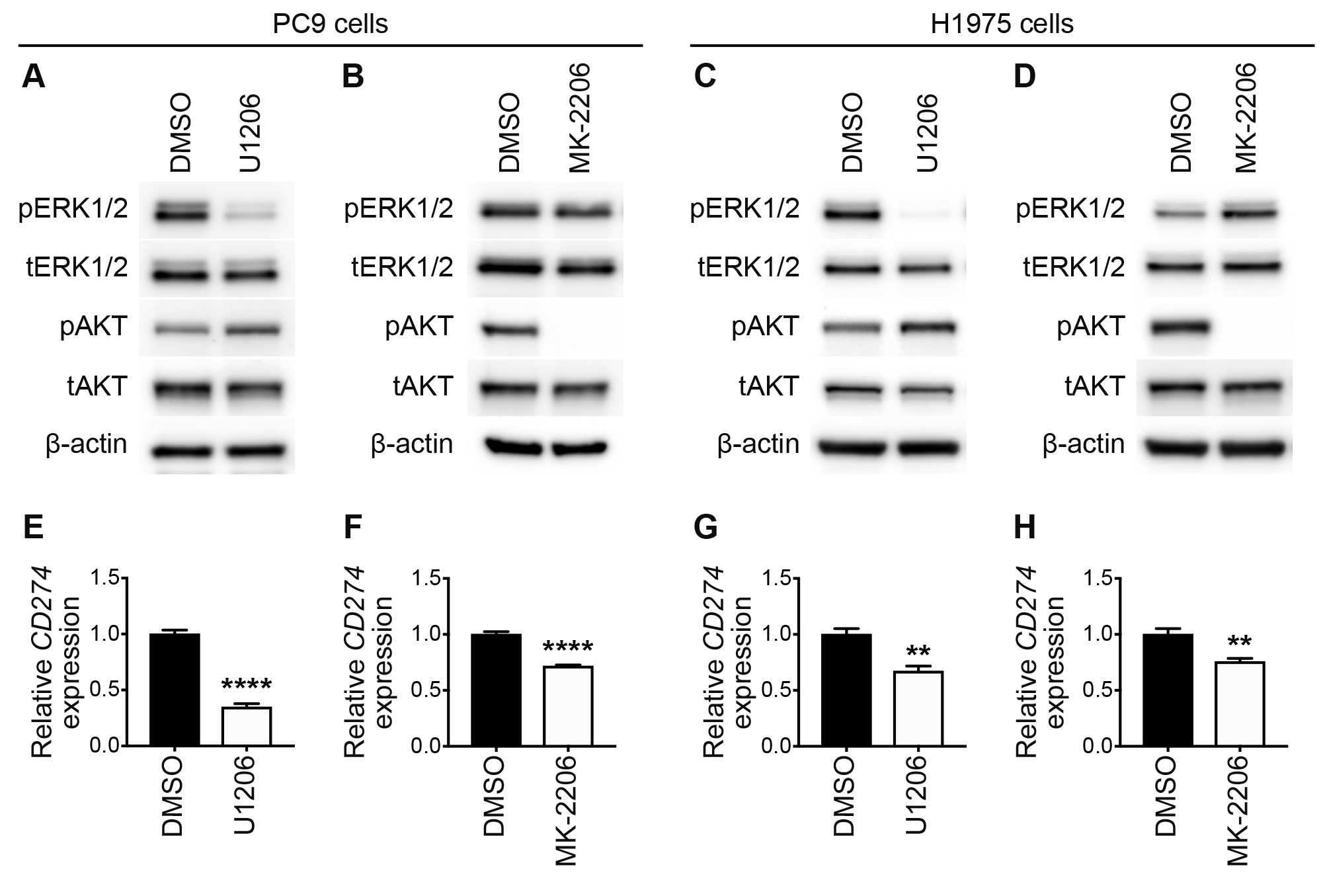
**

**
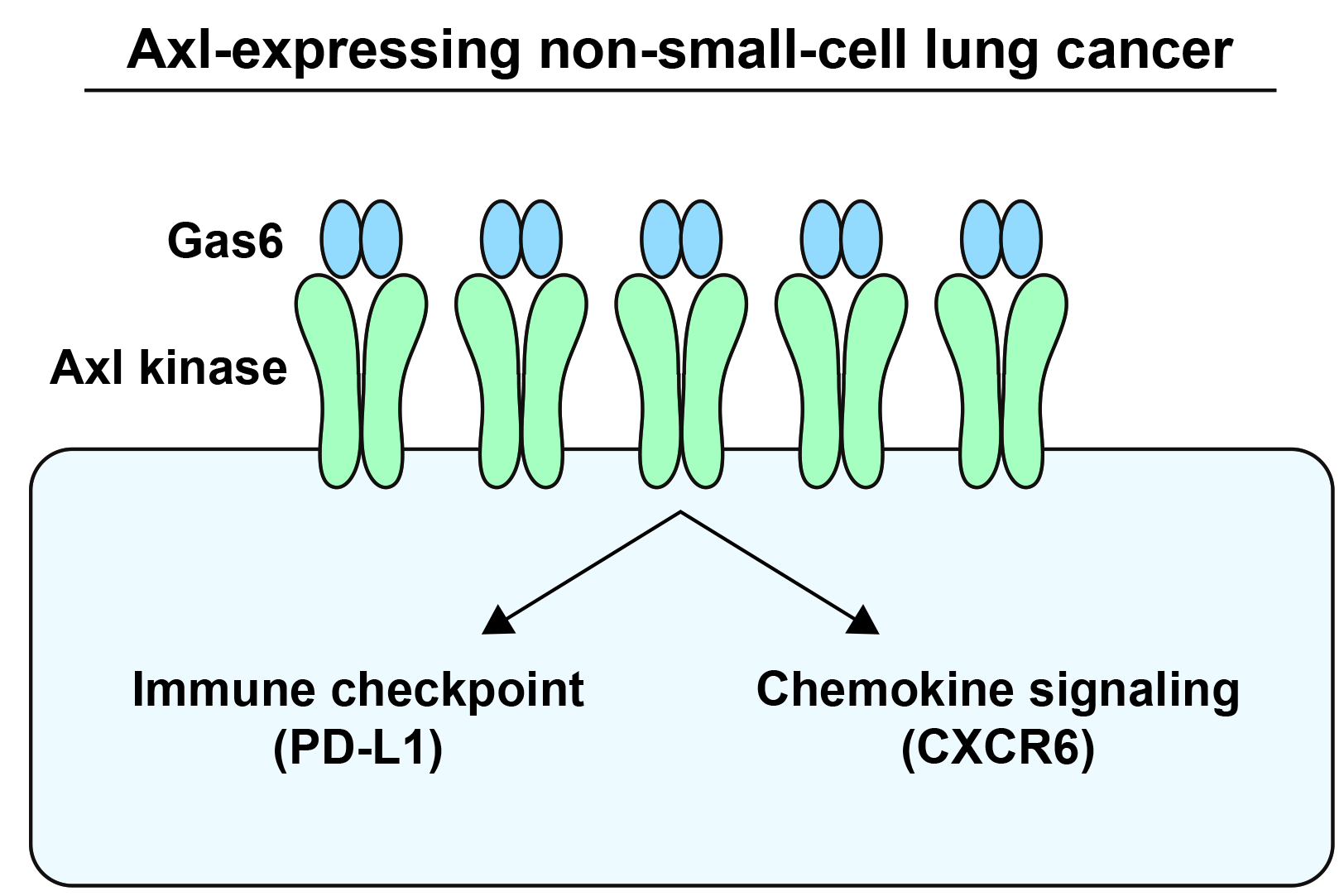
Figure S5:** Diverse downstream pathways driven by Axl receptor tyrosine kinase in non-small-cell lung cancer (NSCLC). Gas6 is a ligand for Axl kinase. Mitogen-activated protein kinases (MAPK) and the epithelial-to-mesenchymal transition (EMT) are known to be involved in proliferation and metastasis respectively. Our data provide the novel role of Axl kinase in up-regulation of genes encoding immune checkpoint molecules (*e.g.* programmed death-ligand1, PD-L1) and chemokine receptors (*e.g.* CXC-receptor 6, CXCR6) in NSCLC.
